# Association of a dysbiotic oral microbiota with the development of focal lymphocytic sialadenitis in IκB-ζ-deficient mice

**DOI:** 10.1038/s41522-020-00158-4

**Published:** 2020-10-30

**Authors:** Junho Lee, Jehan Alam, Eunji Choi, Yeon Kyeong Ko, Ahreum Lee, Youngnim Choi

**Affiliations:** 1 Department of Immunology and Molecular Microbiology, School of Dentistry and Dental Research Institute, Seoul National University, Seoul, Korea; 2 Present Address: Department of Pathology, Asan Medical Center, University of Ulsan College of Medicine, Seoul, South Korea; 3 Present Address: Department of Ophthalmology, Baylor College of Medicine, Houston, TX 77030 USA

**Keywords:** Dentistry, Microbiome, Microbiota

## Abstract

Mice lacking IκB-ζ, a protein encoded by the *Nfkbiz* gene, spontaneously develop a Sjögren’s syndrome-like disease involving the lachrymal glands, but no salivary gland symptoms have been reported. We found that *Nfkbiz*^*−/−*^ female mice presented a significantly reduced salivary flow rate, focal lymphocytic sialadenitis (FLS), and a dysbiotic oral microbiota at week 24. To dissect the contributions of genetic and environmental factors to the salivary gland phenotype, *Nfkbiz*^+/+^ and *Nfkbiz*^−/−^ mice were cohoused after weaning and evaluated at week 20. Cohousing alleviated the salivary gland phenotype of *Nfkbiz*^*−/−*^ mice but did not induce any disease phenotype in *Nfkbiz*^+/+^ mice. Additionally, the oral microbiota in the cohoused mice was synchronized toward that in *Nfkbiz*^+/+^ mice. In conclusion, IκB-ζ-deficient mice developed hyposalivation and FLS, in which a dysbiotic oral microbiota played an important role. This finding suggests that the dysbiotic oral microbiota could be a therapeutic target.

## Introduction

Sjögren’s syndrome (SS) is a heterogeneous autoimmune disease characterized by dryness of the mouth and eyes^1^. Together with reduced saliva/tear secretion, lymphocytic infiltration of the salivary or lacrimal glands and the presence of autoantibodies, particularly those against the SS antigen A/Ro, are important diagnostic criteria for SS^2^. The etiopathogenetic mechanisms of SS remain poorly understood, but it is believed that both genetic and environmental factors are involved in the development of the disease^3^.

Animal models are useful for studying the pathogenesis of SS. Among the several animal models for SS, mice lacking IκB-ζ, a protein encoded by the *Nfkbiz* gene, spontaneously develop an SS-like autoimmune disease from a single genetic defect. The phenotype of SS-like inflammation in *Nfkbiz*^−/−^ mice includes lymphocyte-infiltrated dacryoadenitis, reduced tear secretion, and autoantibodies against the SS antigens A/Ro and B/La^4^. SS-like inflammation depends on IκB-ζ deficiency in epithelial cells but not IκB-ζ deficiency in lymphocytes^4^. IκB-ζ is an inducible regulator of nuclear factor-κB (NF-κB), which positively or negatively modulates the transcriptional activity of NF-κB in a gene-specific manner^5^. IκB-ζ-deficient lachrymal gland epithelial cells present increased apoptosis^4^, a phenomenon that is also observed in the labial salivary glands of SS patients^6,7^.

Although a previous study reported no symptoms involving the salivary glands of *Nfkbiz*^−/−^ mice^4^, we noticed reduced salivary secretion in our colony. It is well known that animal microbiomes significantly affect the immune function and disease phenotype of laboratory animals^8^. Dysbiosis of the oral microbiota in SS patients has been reported^9–12^. However, van den Meulen et al. reported that the changes in the oral microbiota are attributed more to the reduced salivary secretion than to SS^11^, and if the dysbiotic oral microbiota plays any role in the pathogenesis of SS is not clear. Mice have quite simple oral bacterial communities^13^, which is a merit for studying the role of the oral microbiome in the pathogenesis of SS. The fact that the oral microbiota can be examined before the onset of symptoms is another benefit of such animal models. IκB-ζ is induced by various microbial products, and subsequent regulation of the transcriptional activity of NF-κB may play a role in the maintenance of a homeostatic balance between the host and microbiome^14^. Indeed, dysbiosis of the skin microbiome in *Nfkbiz*^−/−^ mice has been reported previously in association with dermatitis^15^. This study aimed to characterize the salivary gland phenotype of IκB-ζ-deficient mice and to investigate its association with the oral microbiota. Cohousing is a widely accepted method to synchronize microbial communities^16^. In the present study, the contributions of genetic and environmental factors to the oral microbiota and salivary gland phenotype were investigated by housing *Nfkbiz*^+/+^ and *Nfkbiz*^−/−^ mice with mice of the same or different genotypes.

## Results

### Distinct salivary gland phenotype and oral dysbiosis in Nfkbiz^−/−^ mice

**Fig. 1:**
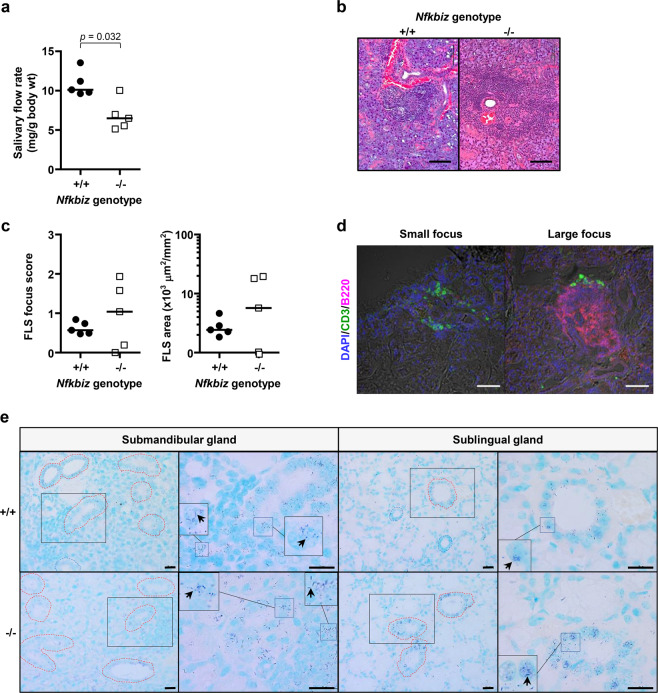
Distinct salivary gland phenotype of Nfkbiz^−/−^ mice. *Nfkbiz*^+/+^ and *Nfkbiz*^−/−^ female mice were kept in separate cages after weaning and were analyzed at 24 weeks. **a** The salivary flow rate was measured after the injection of pilocarpine. Cross bars in the graph represent the median. *P* value was determined by the Mann–Whitney U-test. **b** Representative images of H&E-stained sections of the submandibular glands from *Nfkbiz*^+/+^ and *Nfkbiz*^−/−^ mice. Scale bars, 100 μm. **c** Focal lymphocytic sialadenitis (FLS) score and area expressed as the number of foci per 4 mm^2^ and μm^2^/mm^2^, respectively, were calculated by counting the number of foci and measuring the size of each section. **d** Representative images of FLS foci stained with anti-CD3 and anti-B220 antibodies. Scale bars, 100 μm. **e** The sections of the salivary glands were subjected to in situ hybridization (ISH) using a universal probe. Representative areas with bacterial infection are shown. Arrows indicate representative ISH signals in violet color and the shape of rod or cocci. Red dotted line, duct with bacterial infection. Blue dotted line, duct without infection. Scale bars, 20 μm.

Because an effect of gender on exocrine gland inflammation in mouse models of SS has been reported^17^, only female mice were included in the study. NOD mice, the most commonly used model for SS, develop focal lymphocytic sialadenitis (FLS) before 17 weeks of age and exhibit a decrease in the salivary flow rate between 17 weeks and 24 weeks of age^18^. Therefore, the salivary gland phenotype of *Nfkbiz*^*−/−*^ mice was examined at 24 weeks of age. In contrast to a previous report^4^, significantly reduced salivary secretion was observed in 24-week-old *Nfkbiz*^*−/−*^ mice (Fig. 1a). A small degree of FLS was found in both genotypes. Although there was no significant difference between the groups, three of five *Nfkbiz*^*-/-*^ mice developed FLS with a score ≥ 1, meeting the diagnostic criterion for SS (Fig. 1b, c). Immunofluorescent staining confirmed that infiltrating lymphocytes were mostly T cells in small foci but predominantly B cells in large foci (Fig. 1d), as reported in SS^19^. We previously reported infection of ductal cells and the area of FLS with bacteria in the labial salivary glands obtained from SS patients^20^. In situ hybridization using a 16S rRNA-targeted probe revealed the presence of infected ducts not only near the area of FLS but also at the area without infiltration in both genotypes (Fig. 1e), the degree of which varied depending on the area and mouse.

**Fig. 2:**
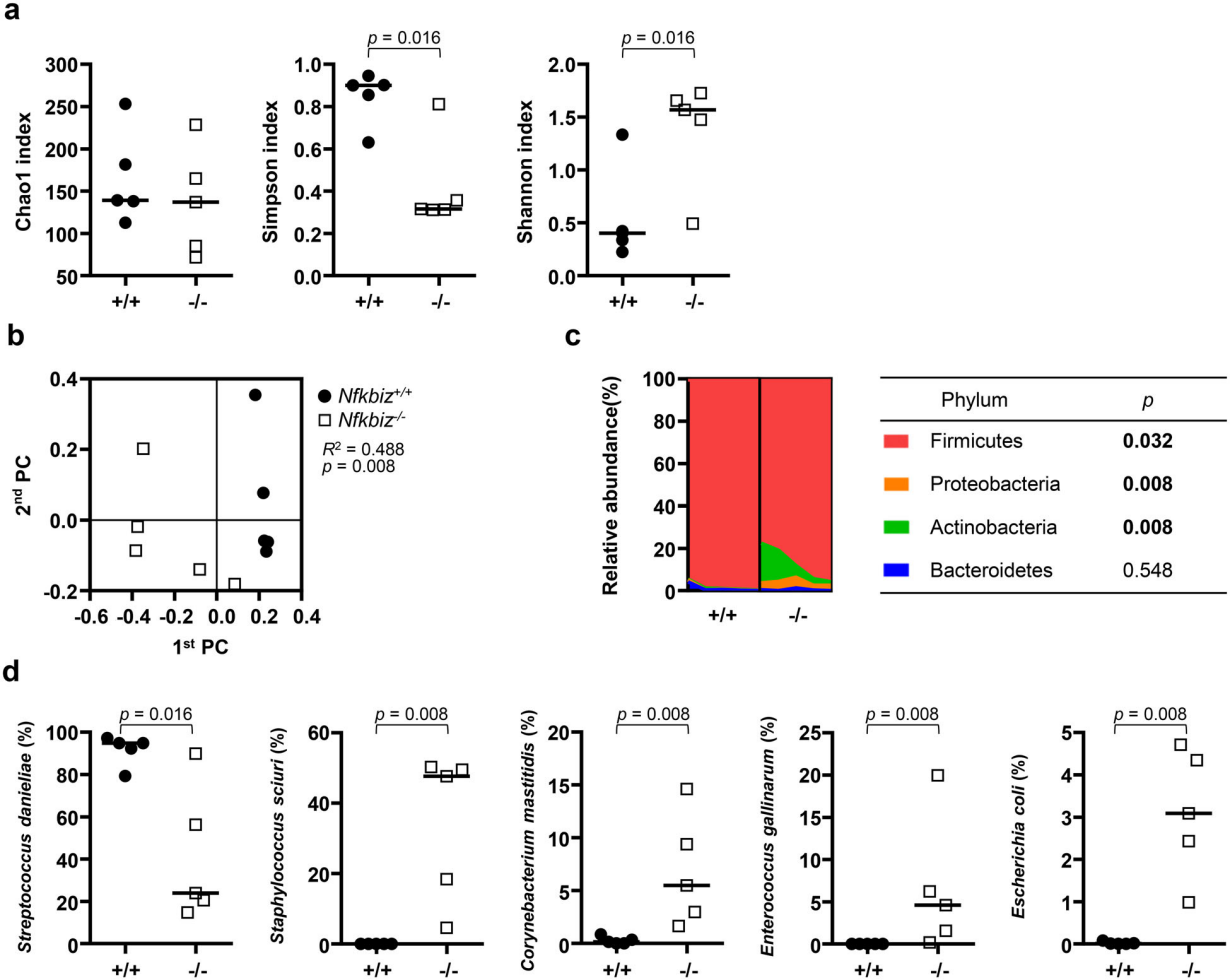
Dysbiosis of the oral microbiota in Nfkbiz^−/−^ mice. Oral bacterial communities collected using cotton swabs were analyzed by high-throughput sequencing of the 16S rRNA gene. **a** The species richness, evenness, and diversity of communities were estimated by the Chao1, Simpson, and Shannon indexes, respectively. **b** Principal coordinates analysis (PCoA) was performed using the generalized UniFrac distance metric with normalization for read counts. *P* value was determined by permutational multivariate analysis of variance (PERMANOVA). **c** Composition of the oral microbiota at the phylum level. Phyla with mean relative abundances ≥ 1% are shown. **d** Relative abundances of the top five species. *P* values were determined by the Mann–Whitney *U*-test. None of the *P* values passed the Benjamini–Hochberg correction.

When the structure and composition of the oral microbiota were analyzed by high-throughput sequencing of the 16S rRNA gene, the estimated species richness using the Chao1 index did not differ between genotypes. However, the species evenness determined using the Simpson index and the Shannon index, which accounts for both richness and evenness, were significantly increased in *Nfkbiz*^*−/−*^ mice compared with those in *Nfkbiz*^+/+^ mice (Fig. 2a). A principal coordinates analysis (PCoA) plot obtained based on the generalized UniFrac distances revealed segregation of oral bacterial communities according to the *Nfkbiz* genotype, and the existence of a significant intergroup difference was confirmed by the permutational multivariate analysis of variance (PERMANOVA) test (*R*^2^ = 0.488, *P* = 0.008, Fig. 2b).

The composition of the oral microbiota also showed differences according to the *Nfkbiz* genotype. At the phylum level, compared with *Nfkbiz*^+/+^ mice, *Nfkbiz*^*−/−*^ mice showed increases in the relative abundances of Actinobacteria and Proteobacteria at the expense of Firmicutes (Fig. 2c). At the species level, the oral bacterial communities of *Nfkbiz*^+/+^ mice were dominated by *Streptococcus danieliae* (Supplementary Fig. 1), which accounted for up to 97% of the total bacteria. The dominance of *S. danieliae* significantly decreased; instead, the relative abundances of *Staphylococcus sciuri, Escherichia coli, Enterococcus gallinarum*, and *Corynebacterium mastitidis* increased in *Nfkbiz*^*−/−*^ mice (Fig. 2d).

Collectively, the results showed that *Nfkbiz*^*−/−*^ mice presented the salivary gland phenotype of SS and an altered oral microbiota at 24 weeks of age.

### Unidirectional effect of cohousing on the salivary gland phenotype

**Fig. 3:**
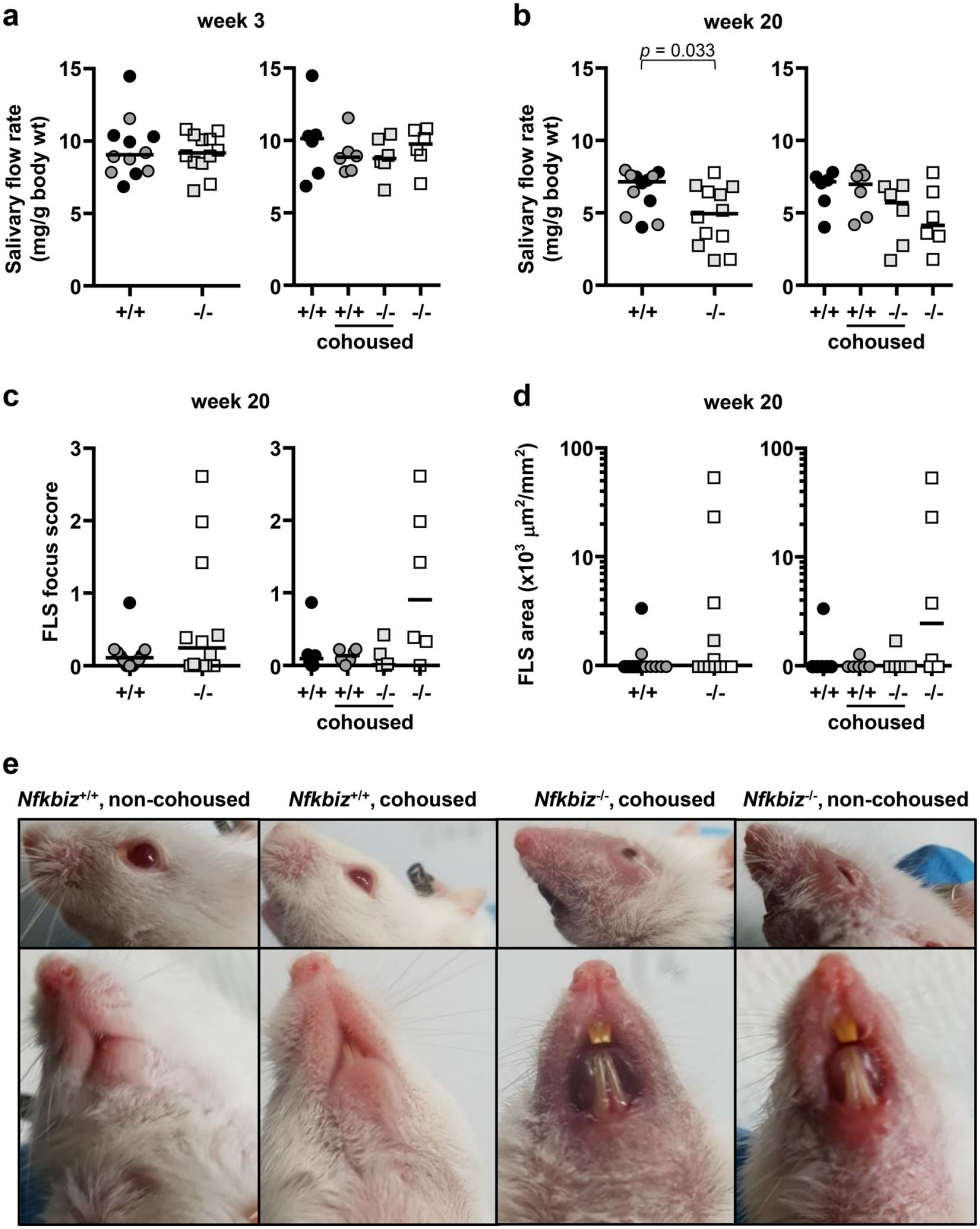
Unidirectional effect of cohousing on the salivary gland phenotype of Nfkbiz^−/−^ mice. *Nfkbiz*^*+/+*^ and *Nfkbiz*^*−/−*^ female mice were randomly divided into two groups, namely, non-cohoused or cohoused, at week 3 and were analyzed at week 20. **a** The salivary flow rate was measured after the injection of pilocarpine at week 3 before cohousing. **b** The salivary flow rates were measured again at week 20. **c** Focal lymphocytic sialadenitis (FLS) scores expressed as the number of foci per 4 mm^2^ were calculated by counting the number of foci and measuring the size of each section. **d** FLS areas were calculated by measuring the sizes of foci and each section. **a**–**d** Data are presented by genotype (left) or by genotype and housing condition (right). Crossbars in the graphs represent the median. *P* value was determined by the Mann–Whitney *U*-test. **e** The periocular and perioral inflammation in 20-week-old *Nfkbiz*^*−/−*^ mice was photographed.

To dissect the contributions of genetic and environmental factors to the oral microbiota and salivary gland phenotype, the *Nfkbiz*^+/+^ and *Nfkbiz*^−/−^ mice were randomly divided into two groups (*n* = 6 per group) upon weaning at week 3, housed with mice of the same or different genotype, and evaluated at week 20 (Supplementary Table 1). The salivary flow rate was not different according to the genotype at week 3 but was significantly reduced in *Nfkbiz*^−/−^ mice at week 20 when the mice from different housing conditions were combined. When the mice were divided into four groups by genotype and housing condition, there was no significant intergroup difference (*P* = 0.206). However, while the median salivary flow rate of the *Nfkbiz*^+/+^ mice was not changed by cohousing, that of the *Nfkbiz*^−/−^ mice was slightly increased by cohousing (Fig. 3a, b). Similarly, cohousing affected the FLS score of *Nfkbiz*^*−/−*^ mice but not that of *Nfkbiz*^+/+^ mice: while three of the noncohoused *Nfkbiz*^*−/−*^ mice developed FLS with a score ≥ 1 at week 20, none did so among the other three groups, resulting in a significant difference in its incidence (*χ*^2^ = 6.371, *P* = 0.04 by Fisher’s exact test, Fig. 3c). The FLS area presented a similar pattern as the FLS score (Fig. 3d). Interestingly, the disease phenotype that appeared in the periocular and perioral areas^4^ was not changed by cohousing (Fig. 3e). Collectively, the results showed that the salivary gland phenotype of *Nfkbiz*^*−/−*^ mice was alleviated by cohousing with *Nfkbiz*^+/+^ mice, but this cohousing did not induce any phenotype in *Nfkbiz*^+/+^ mice.

### Asymmetrical effect of cohousing on the oral microbiota

To investigate the effect of cohousing on the oral microbiota, bacterial communities collected at weeks 3 and 20 were analyzed. From the unidirectional effect of cohousing on the salivary gland phenotype, we expected that cohousing would affect the oral microbiota of *Nfkbiz*^*−/−*^ mice but not that of *Nfkbiz*^+/+^ mice.

**Fig. 4:**
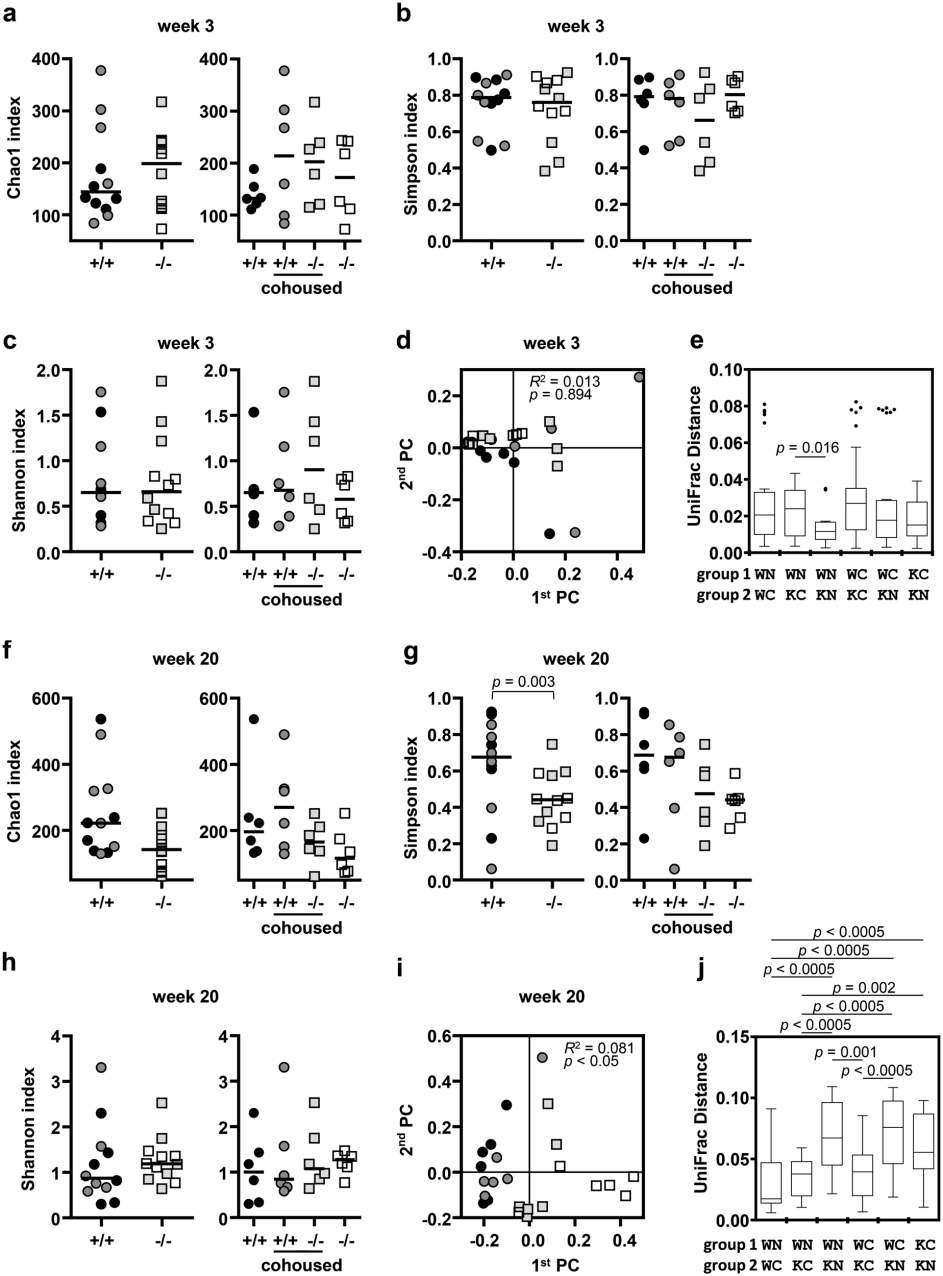
Asymmetrical effect of cohousing on the structure of the oral microbiota. Oral bacterial communities collected at weeks 3 and 20 were analyzed by high-throughput sequencing of the 16S rRNA gene. Diversities at week 3 (**a**–**e**) and week 20 (**f**–**j**) are presented. The species richness, evenness, and diversity of communities were estimated by the Chao1 (**a**, **f**), Simpson (**b**, **g**), and Shannon (**c**, **h**) indexes, respectively. Data are presented by genotype (left) or by genotype and housing condition (right). Crossbars in the graph represent the median. *P* value was determined by the Mann–Whitney *U*-test. Principal coordinates analysis (PCoA) was performed using the generalized UniFrac distance metric (**d**, **i**). *P* values were determined by PERMANOVA. Intergroup UniFrac distances are presented with box-and-whisker plots in which a box is drawn from the first quartile, median, and third quartile, and whiskers are drawn from the minimum and maximum (**e**, **j**). Several outliers are presented as dots. WN wild-type_noncohoused, WC wild-type_cohoused, KN knock-out_noncohoused, KC knock-out_cohoused. *P* values were determined by the Kruskal–Wallis test followed by the Bonferroni post hoc method.

At week 3, there were no intergroup differences in any alpha diversity index and no clear segregation of the communities among groups in the PCoA plot obtained based on the generalized UniFrac distances (*R*^*2*^ = 0.013, *P* = 0.894, Fig. 4a–d). Although mice were randomly assigned to housing conditions, the intergroup distance analysis revealed that the communities of noncohoused *Nfkbiz*^+/+^ and *Nfkbiz*^*−/−*^ mice were more similar to each other than to those of the cohoused mice at week 3 (Fig. 4e). At week 20, the Simpson index was significantly reduced in *Nfkbiz*^*−/−*^ mice, as observed in 24-week-old mice. However, the increase in the Shannon index in *Nfkbiz*^*−/−*^ mice observed in 24-week-old mice was not recapitulated at week 20 (Fig. 4f–h). In the PCoA plot, the clear segregation of the communities of noncohoused *Nfkbiz*^*−/−*^ mice from those of noncohoused *Nfkbiz*^+/+^ mice was recapitulated. While the communities of cohoused *Nfkbiz*^+/+^ mice were clustered together with those of noncohoused *Nfkbiz*^+/+^ mice, the communities of cohoused *Nfkbiz*^*−/−*^ mice clustered away from those of the noncohoused *Nfkbiz*^*−/−*^ mice toward those of the cohoused *Nfkbiz*^+/+^ mice (*R*^*2*^ = 0.081, *P* < 0.05, Fig. 4i). The intergroup distances confirmed that the communities of the *Nfkbiz*^*−/−*^ mice became similar to those of the noncohoused and cohoused *Nfkbiz*^+/+^ mice after cohousing but different from those of *Nfkbiz*^+/+^ mice in the non-cohoused group (Fig. 4j).

**Fig. 5:**
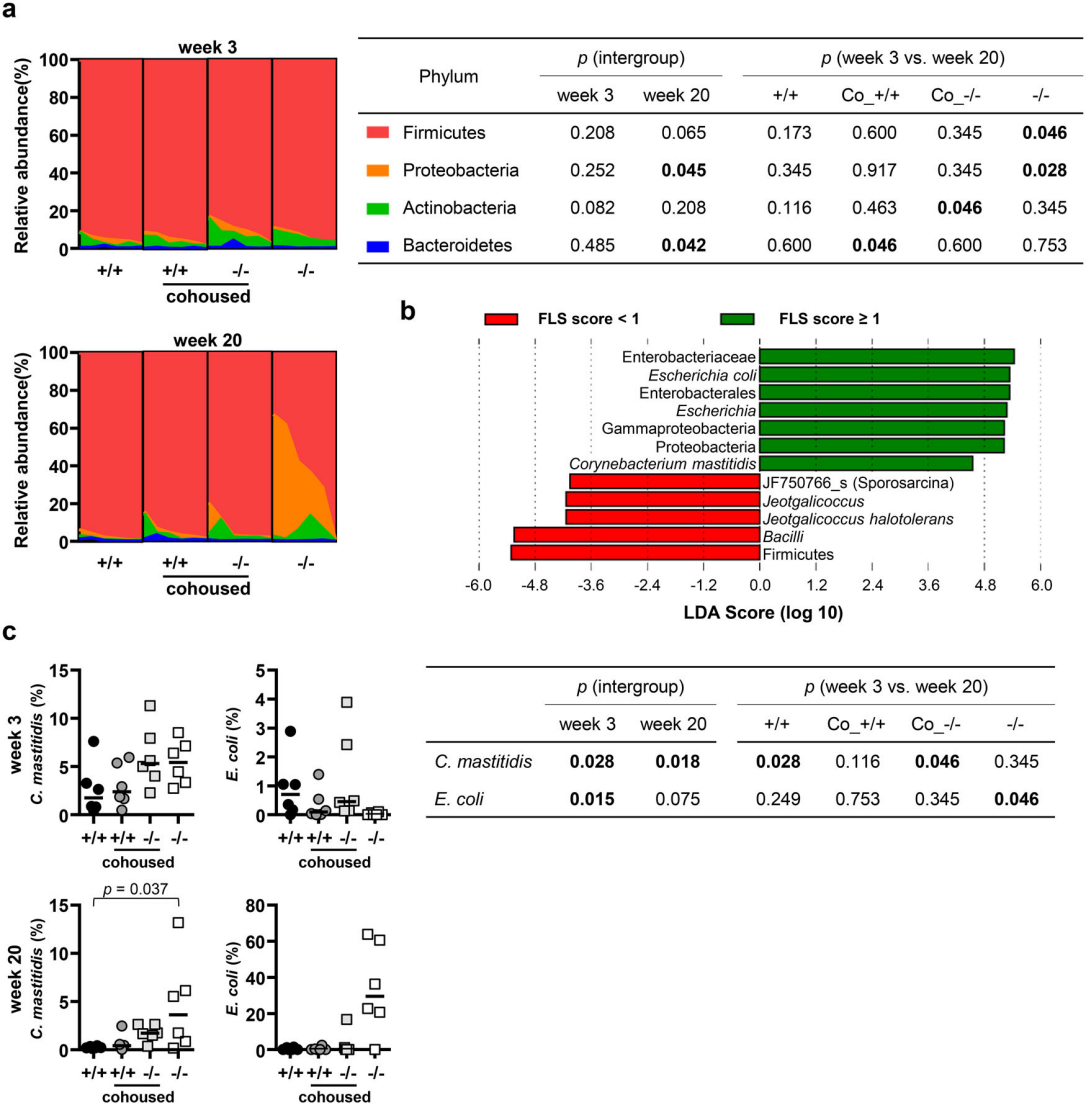
Asymmetrical effect of cohousing on the composition of the oral microbiota. Oral bacterial communities collected at weeks 3 and 20 were analyzed by high-throughput sequencing of the 16S rRNA gene. **a** The compositions of phyla with mean relative abundances ≥ 1% at weeks 3 and 20 are presented. *P* values for intergroup differences were obtained by the Kruskal–Wallis test. *P* values for differences between weeks 3 and 20 were obtained by Wilcoxon’s signed rank test. **b** Taxa associated with FLS score ≥ 1 were determined by linear discriminant analysis effect size (LEfSe) analysis. **c** The relative abundances of species associated with FLS score ≥ 1 at weeks 3 and 20 are depicted. Crossbars in the graphs represent the median. *P* values for intergroup differences were obtained by the Kruskal–Wallis test. *P* values for differences between weeks 3 and 20 were obtained by the Wilcoxon signed rank test. None of the *P* values passed the Benjamini–Hochberg correction.

The composition of the oral microbiota at the phylum level also clearly revealed the asymmetrical effect of cohousing on the oral microbiota. At week 3, *S. danieliae* was the most abundant species in 23 of 24 mice. Proteobacteria was significantly enriched at the expense of Firmicutes in the noncohoused *Nfkbiz*^*−/−*^ mice from week 3 to week 20. Such *Nfkbiz*^*−/−*^ genotype-specific changes were not observed in the *Nfkbiz*^*−/−*^ mice cohoused with *Nfkbiz*^+/+^ mice. The enrichment of Bacteroidetes and the depletion of Actinobacteria over the cohousing period observed in the *Nfkbiz*^+/+^ and *Nfkbiz*^*−/−*^ mice, respectively, reflected the characteristics of the *Nfkbiz*^+/+^ communities (Fig. 5a).

To analyze associations between the salivary gland phenotype and oral microbiota, we chose only FLS because there was no adequate criterion for oral dryness based on the salivary flow rate induced with pilocarpine. A linear discriminant analysis effect size (LEfSe) analysis revealed several taxa associated with FLS score ≥ 1, including *C. mastitidis* and *E. coli* (Fig. 5b). The relative abundance of *C. mastitidis* was already higher in *Nfkbiz*^*−/−*^ mice than in *Nfkbiz*^+/+^ mice at week 3 (median 5.3 vs. 1.8, *P* = 0.002). In the noncohoused *Nfkbiz*^*-/-*^ mice, the high relative abundance of *C. mastitidis* was maintained, and *E. coli* was enriched at week 20. In the cohoused *Nfkbiz*^*−/−*^ mice, however, the relative abundances of both *C. mastitidis* and *E. coli* were synchronized toward those in *Nfkbiz*^+/+^ mice (Fig. 5c).

Collectively, the results showed the following findings. First, the oral microbiota of non-cohoused *Nfkbiz*^*−/−*^ mice was not different from that of *Nfkbiz*^*+/+*^ mice at week 3 but developed dysbiosis, which we specifically define as the loss of *S. danieliae*-predominated structure and the enrichment of FLS-associated taxa, at week 20. Second, cohousing of *Nfkbiz*^*+/+*^ and *Nfkbiz*^*−/−*^ mice synchronized their oral microbiota toward that of *Nfkbiz*^*+/+*^ mice.

### Increased J chain expression and salivary IgA (sIgA) secretion in Nfkbiz^−/−^ mice

**Fig. 6:**
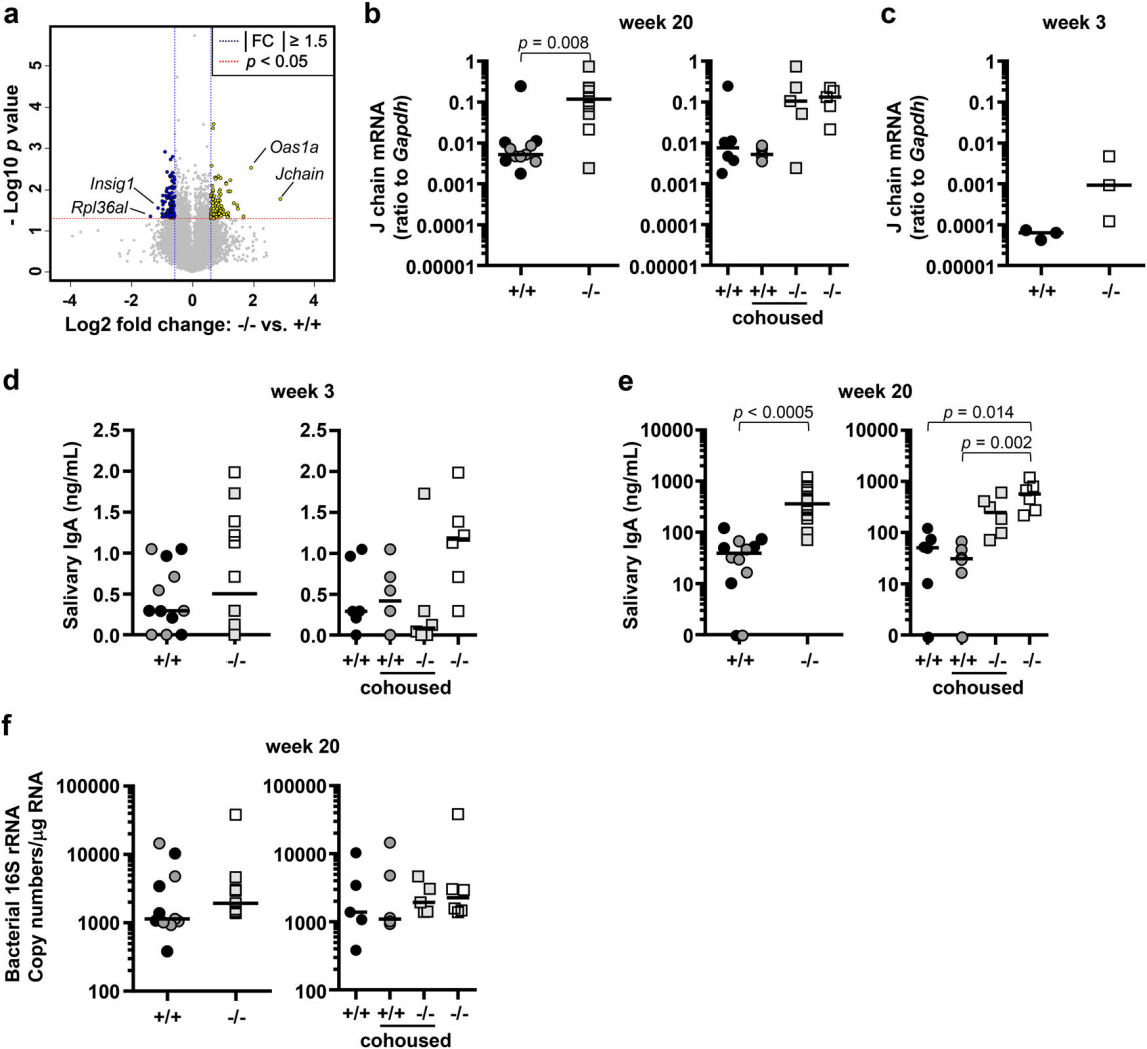
Increased Jchain expression and salivary IgA secretion in Nfkbiz^−/−^ mice. Saliva and salivary glands were used to measure the levels of sIgA and *Jchain*, respectively. **a** RNA samples extracted from the salivary glands of 20-week-old noncohoused *Nfkbiz*^+/+^ and *Nfkbiz*^−/^^−^ mice (*n* = 2 per group) were subjected to whole-transcriptome analysis using microarrays. The results are presented as a volcano plot. **b** Expression levels of *Jchain* mRNA in the salivary glands at week 20 were determined by qRT-PCR. **c** Expression levels of *Jchain* mRNA in the salivary glands obtained from six additionally sacrificed 3-week-old mice were determined by qRT-PCR. **d**, **e** The concentrations of sIgA in saliva samples collected at weeks 3 and 20 were measured by ELISA. **f** Expression levels of bacterial 16S rRNA in the salivary glands at week 20 were determined by qRT-PCR. Crossbars in the graphs represent the median. P values were determined by the Mann–Whitney U-test and Kruskal–Wallis test followed by post hoc Bonferroni correction.

We thought that the dysbiosis of the oral microbiota observed in *Nfkbiz*^*−/−*^ mice may be attributed to dysregulated production of antimicrobial peptides or proteins by the salivary glands. Microarray analysis of the salivary glands obtained from the 20-week-old noncohoused *Nfkbiz*^+/+^ and *Nfkbiz*^−/−^ mice (*n* = 2 per group) revealed 212 differentially expressed genes (DEGs): 107 upregulated and 105 downregulated in *Nfkbiz*^−/−^ mice compared with *Nfkbiz*^+/+^ mice. The DEGs included a number of genes involved in immune responses, apoptosis, and autophagy. Only one gene, *Jchain*, which encodes the joining chain for secretory IgA and IgM, was associated with antimicrobial function and presented the highest fold change (Fig. 6a and Supplementary Table 2). Although none of the DEGs passed the Benjamini–Hochberg test for the correction of multiple comparisons, quantitative reverse transcription polymerase chain reaction (qRT-PCR) confirmed the significantly higher levels of *Jchain* expression in *Nfkbiz*^−/−^ mice than in *Nfkbiz*^+/+^ mice, and the levels were barely affected by cohousing (Fig. 6b). To further clarify whether the increased *Jchain* expression was caused by the dysbiotic oral microbiota, the expression of *Jchain* was examined in the salivary glands obtained from 3-week-old *Nfkbiz*^+/+^ and *Nfkbiz*^−/−^ mice that were additionally sacrificed. The levels of *Jchain* expression were substantially lower at week 3 than at week 20, but increased *Jchain* expression in *Nfkbiz*^−/−^ mice started to appear at week 3 (Fig. 6c). Since *Jchain* expression indicates the presence of plasma cells, the levels of sIgA, the major Ig isotype in saliva, were determined by ELISA. At week 3, the levels of sIgA were very low in all mice, with no intergroup difference (Fig. 6d). At week 20, the levels of sIgA were much higher in *Nfkbiz*^−/−^ mice than in *Nfkbiz*^+/+^ mice, which was significant only in the noncohoused group, suggesting the presence of a cohousing effect (Fig. 6e). Correlation analyses between the levels of sIgA and the structure and composition of the oral microbiota revealed a moderate negative correlation with the Simpson index and negative or positive correlations with several species, including *S. danieliae* (Table 1).

**Table 1:** Characteristics of the oral microbiota that are significantly correlated with the levels of salivary IgA.

	Spearman’s rho	*P*^a^	*q*^b^
Simpson	−0.459	0.001	0.017
*Streptococcus parasanguinis* group	−0.567	<0.0005	0.001
*Streptococcus sinensis* group	−0.564	<0.0005	0.001
*Lactobacillus murinus*	−0.471	0.001	0.014
*Streptococcus danieliae*	−0.445	0.002	0.022
*Enterococcus gallinarum*	0.588	<0.0005	0.001

^a^Spearman’s rank correlation test.

^b^Corrected by the Benjamini–Hochberg procedure.

Because the bacterial infection of the salivary glands observed by in situ hybridization in the 24-week-old mice was difficult to quantify, the degree of bacterial infection was examined by qRT-PCR. Although the median copy numbers of bacterial 16S rRNA was slightly higher in *Nfkbiz*^-/-^ mice than in *Nfkbiz*^+/+^ mice, the difference was not significant (*P* = 0.171 by Mann–Whitney test), and the levels were not affected by cohousing (Fig. 6f).

## Discussion

This study presented the role of a dysbiotic oral microbiota in the development of a reduced salivary flow rate and FLS in *Nfkbiz*^−/−^ mice, a model for SS: while the noncohoused *Nfkbiz*^−/−^ mice developed dysbiosis of the oral microbiota and a salivary gland phenotype, synchronization of the oral microbiota toward that of *Nfkbiz*^+/+^ mice through cohousing ameliorated the phenotype. A couple of reasons can be proposed for the lack of obvious salivary gland symptoms found in a previous study^4^. Although details regarding the housing conditions of the mice were not described, if *Nfkbiz*^−/−^ mice and their littermate controls used in the previous study were housed together, the *Nfkbiz*^−/−^ mice would not have developed the symptoms. In addition, the previous study used both male and female mice, but we found in our preliminary work that male *Nfkbiz*^−/−^ mice do not develop severe FLS. Similarly, in NOD mice, males develop far worse dacryoadenitis, whereas females develop far more severe FLS^17^.

This study provided strong evidence for the control of the oral microbiota by host genetics. The wild-type mice had very simple oral bacterial communities dominated by *S. danieliae* that comprised up to 97% of the total bacteria. This observation is consistent with our previous report showing that relatively simple murine oral bacterial communities of C57BL/6 mice are dominated by a tentative *Streptococcus* species represented by the GenBank accession no. EU453973^13^. The 16S rRNA sequence of EU453973 is identical to that of *S. danieliae*, which was later isolated from the mouse cecum^21^. The predominance of *S. danieliae* substantially decreased in the noncohoused *Nfkbiz*^−/−^ mice, increasing the species evenness. Although some of the 24-week-old mice were not littermates, oral bacterial communities were shown to cluster depending on the *Nfkbiz* genotype. Repeated examination at weeks 3 and 20 revealed that the oral bacterial communities of *Nfkbiz*^+/+^ and *Nfkbiz*^−/−^ mice were segregated away from each other over time. All these findings suggest the strong influence of IκB-ζ on the oral microbiota. Most taxa that were enriched in *Nfkbiz*^−/−^ mice were also found in *Nfkbiz*^+/+^ mice at very low abundances. This result suggests that the lack of IκB-ζ leads to the expansion of indigenous species rather than colonization by new species. The observation that there was no increase in species richness but an increase in species evenness also supports this contention.

Several potential mechanisms for the changes in the oral microbiota caused by IκB-ζ deficiency can be suggested. Increased apoptosis in IκB-ζ-deficient epithelial cells may have changed the colonization niche in the oral cavity. Changes in the levels of NF-κB-driven immune mediators, such as cytokines and antimicrobial peptides, in the oral mucosa may have differentially affected the fitness of species. Interestingly, the levels of sIgA were significantly higher in the noncohoused *Nfkbiz*^*−/−*^ mice than in *Nfkbiz*^*+/+*^ mice. Secretory IgA plays a critical role in shaping the microbiota in the gut and oral cavity: it can prevent, alter, or promote colonization of mucosal tissues^22–24^. Alternatively, the ability to induce sIgA could differ depending on taxa, as periodontitis patients have increased levels of sIgA^22^. *Nfkbiz*^−/−^ mice had a propensity to express increased levels of *Jchain* in the salivary glands before the onset of dysbiosis, suggesting the possibility that increased sIgA drives dysbiosis. On the other hand, the lack of a significant increase in sIgA levels in the cohoused *Nfkbiz*^−/−^ mice suggests the possibility that the dysbiotic microbiota contributed to the increased sIgA levels. The role of sIgA in the establishment and maintenance of the oral microbiota awaits further clarification. Environmental factors can also be considered. Since oral dryness has a tremendous effect on the composition of the oral microbiota^11^, hyposalivation could have contributed to the altered oral microbiota of the noncohoused *Nfkbiz*^−/−^ mice. In addition, severe inflammation around the perioral skins hindered complete closure of the mouth in *Nfkbiz*^*−/−*^ mice, which could have affected the environment of the oral cavity.

Importantly, the taxa enriched in the noncohoused *Nfkbiz*^−/−^ mice were associated with FLS scores ≥ 1. Among the FLS-associated taxa, *C. mastitidis* was first isolated from the milk of sheep with subclinical mastitis and was detected in the preputial gland abscesses of mice^25,26^. It has also been reported as an ocular commensal in mice, protecting them from corneal infection with pathogens by inducing an IL-17 response in mucosal γδ T cells^27^. *Nfkbiz*^*−/−*^ mice have a defect in Th17 development and increased apoptosis in epithelial cells^4,28^. Under such conditions, *C. mastitidis* may contribute to inflammation in the salivary glands. *E. coli* is a common gut commensal of humans and mice, although there are diverse pathogenic strains that can cause diseases in the intestinal or urinary tracts^29^. *E. coli* has also been detected in the oral cavity of humans and mice, and oral strains have been recently isolated from the biopsy tissues of oral lichen planus patients^30,31^. *E. gallinarum*, which was enriched in *Nfkbiz*^*−/−*^ mice, is known to translocate from the gut to the liver and induce autoantibodies in (NZW × BXSB)F1 mice, an autoimmune-prone strain. Interestingly, *E. gallinarum* could not translocate in C57BL/6 mice under pathogen-free conditions but did so under monocolonized conditions^32^. In other words, the commensal microbiota can not only induce but also block autoimmune reactions^33,34^. The *S. danieliae*-predominated oral microbiota, which was lost in the noncohoused *Nfkbiz*^*−/−*^ mice, may be specialized in a tolerant coexistence with the host. Induction of the antigen-presenting cell-like phenotype in human submandibular gland tumor cells by SS-associated bacterial species and infection of ductal cells with bacteria in the labial salivary glands obtained from SS patients have been presented as potential mechanisms for driving FLS^20^. Bacterial infection of the salivary glands was observed in both *Nfkbiz*^*+/+*^ and *Nfkbiz*^*−/−*^ mice. The ability of bacteria to induce cytokines and chemokines from the host cells varies depending on the species^20,35^, which may be also accounted for. Mitigation of FLS in the cohoused *Nfkbiz*^*−/−*^ mice that harbored the *Nfkbiz*^*+/+*^-type oral microbiota supports the role of the dysbiotic oral microbiota in the development of FLS. Further investigations in germ-free and gnotobiotic conditions would answer which arm between the aggressive and protective one has more role in the salivary gland phenotype of *Nfkbiz*^*−/−*^ mice.

The oral bacteria would have been transmitted between the cohoused mice during chewing of the beddings and foods. Interestingly, cohousing asymmetrically affected the oral microbiota of *Nfkbiz*^*+/+*^ and *Nfkbiz*^*−/−*^ mice. The analyses of intergroup distances revealed that the microbiota of *Nfkbiz*^*−/−*^ mice became similar to that of *Nfkbiz*^*+/+*^ mice after cohousing but not vice versa. Asymmetrical microbial transmission between two mouse populations has been reported in a study using C57BL/6 mice from two different vendors^16^. What determines horizontal transmission and the maintenance of a beneficial microbiota is poorly understood but is an important question. In the current study, cohousing of young mice with similar oral microbiotas prevented dysbiosis. Whether the dysbiotic oral microbiota in adult mice can be corrected by cohousing remains to be determined.

Since the cohousing study had to be carried out within the limited animal space and time, the predetermined sample size could not be achieved, and the salivary gland phenotype was evaluated at week 20 instead of week 24. As reported in NOD mice^18^, reduced salivary secretion may follow FLS development. If mice were evaluated at week 24 with an increased sample size, the salivary gland phenotype, particularly the decrease in salivary flow rate, could have been more robust. Another limitation of the current study is the lack of information on the gut microbiota. Gut microbial dysbiosis has also been reported in SS patients particularly in association with dry eyes^12,36^. There is a possibility that the gut microbiota contributed to the development and mitigation of FLS in the noncohoused and cohoused *Nfkbiz*^*−/−*^ mice, respectively, through regulation of the common mucosal immune system.

In conclusion, IκB-ζ-deficient mice developed hyposalivation and FLS, in which the dysbiotic oral microbiota played an important role. This finding suggests that the dysbiotic oral microbiota can be a therapeutic target.

## Methods

### Mouse breeding and sample collection

The experimental protocols and animal handling procedures were approved by the Seoul National University Animal Care and Use Committee (#SNU-180914-8) and conducted in accordance with the Laboratory Animals Act 9025 of the Republic of Korea. *Nfkbiz*^+/−^ mice in the 129/Ola × C57BL/6 background were provided by Professor Shizuo Akira (IFReC, Osaka University, Japan)^28^. All mice were maintained under specific pathogen-free conditions at 18–23 °C with 40–60% humidity and 12 light/12 dark cycle in the Laboratory Animal Facility at the School of Dentistry, Seoul National University. Mice were bred and genotyped at two weeks of age by PCR with a common forward primer 5′-GTTTAAGGTGGCGGCTTCTGCTCTTTG-3′ and the reverse primers 5′- GCTCATCCAGCTAACCTGAACAGTGTT-3′ for the wild-type allele and 5′- CTAAAGCGCATGCTCCAGACTGCCTTG-3′ for the knock-out allele, respectively.

The sample size (*n* = 5 per group) for the initial study using 24-week-old mice was chosen based on a previous study that reported the lachrymal gland phenotype^4^. Using the intergroup distance of the oral microbiota and the incidence of FLS score ≥ 1 obtained in an initial study, the sample sizes for the cohousing study were determined to be *n* = 4 per group to achieve a significant intergroup difference in the oral microbiota based on the simulated PERMANOVA power estimation^37^ and *n* = 8 per group to achieve a significant difference in the incidence of FLS score ≥ 1 at *α* = 0.05 and 80% power.

In the initial study, a mixture of littermates and non-littermates of *Nfkbiz*^+/+^ and *Nfkbiz*^−/−^ mice were housed in separate cages after weaning at 3 weeks of age until sacrifice at week 24. In the cohousing study, since only female mice were used, it was impossible to use littermates for all four groups. Therefore, six breeding sets using mice from the same litter were prepared, and *Nfkbiz*^+/+^ and *Nfkbiz*^−/−^ mice born within a 4-day interval were cohoused after weaning. The mice born within an interval of more than 4 days were housed separately by genotype. The initial sample size for the cohousing study was set as *n* = 8 per group. However, five mice were lost during saliva collection or housing due to mishandling. Two mice were not born within the study period. Finally, all data sets were available only for *n* = 6 per group, and one non-cohoused *Nfkbiz*^−/−^ mouse that was born last was excluded from the analysis.

In the initial study, saliva, oral bacterial samples, and salivary glands were collected at week 24. In the cohousing study, saliva and oral bacterial samples were collected on the day of weaning (week 3) and at week 20. The salivary glands, including the parotid, sublingual, and submandibular glands, were carefully harvested: a half piece was used for histological analysis, and the other half was subjected to RNA extraction using the easy-BLUE^TM^ Total RNA Extraction Kit (iNtRON Biotechnology, Seongnam, Gyeonggi, Korea).

### Saliva collection

Mice anesthetized with isoflurane were intraperitoneally injected with 10 μg/g body weight pilocarpine to stimulate saliva production. Saliva was collected for 10 min after pilocarpine injection and weighed.

### Histological analysis of salivary glands

The salivary glands were fixed with 10% neutral formalin solution (Sigma, St Louis, MO, US) and embedded in paraffin. Sections (4–5 μm) were cut and stained with hematoxylin and eosin (H&E). The FLS score was calculated by counting the number of foci with 50 or more periductal or perivascular mononuclear cells and measuring the sizes of tissue sections and expressed as the number of foci per 4 mm^2^. The area of FLS was also measured.

### Immunofluorescence

A piece of the salivary glands obtained from 24-week-old mice was embedded in OCT compound (Sakura Finetek, Torrance, CA, USA) and frozen in liquid nitrogen. Sections of 10-μm thickness were blocked with 10% horse serum in PBS. The sections were stained with mouse antihuman CD3-ζ mAb clone 6B10.2 (catalog No. sc-1239, 1:75 dilution, Santa Cruz Biotechnology Inc, Dallas, TX, USA) and PE-conjugated rat antimouse B220 clone RA3-6B2 (catalog No. 553090, 1:75 dilution, BD Bioscience, San Jose, CA, USA) followed by goat antimouse IgG (H + L) Alexa 488 catalog No. A28175 (1:1000 dilution, Thermo Scientific, Waltham, MA, USA). Nuclei were stained with DAPI. Immunofluorescence images were captured under a confocal microscope (Leica, Wetzlar, Germany).

### In situ hybridization

A universal probe was prepared by PCR amplification of a 70-bp DNA fragment (5′-CAGGTGCTGCATGGCTGTCGTCAGCTCGTGTTGTGAAATGTTGGGTTAAGTCCCGCAACGAGCGCAACCC-3′), which was synthesized as an oligonucleotide, with the forward (5′-CAG GTR CTG CMT GGY-3′) and reverse (5′-AGG GTT GCG CTC GTT-3′) primers and subsequent labeling with digoxigenin. The sections of the salivary glands were sequentially pretreated with 0.1 N HCl, 1 μg/ml proteinase K, and 0.1 M triethanolamine-HCl and were then hybridized with a digoxigenin-labeled universal probe targeting the 16S rRNA gene. As a negative control, hybridization was performed with the labeled probe with a 10-fold excess amount of nonlabeled probe. The bound probe was detected with alkaline phosphatase-conjugated anti-digoxigenin antibody (Roche, Mannheim, Germany) and visualized with a nitroblue tetrazolium/5-bromo-4-chloro-3-indolyl phosphate solution. The sections were counter stained with methyl green.

### 16S rRNA gene sequencing and microbiota analysis

The oral microbiota was collected from the mouse oral cavity by swabbing the surface of the oral mucosa with cotton swabs. Genomic DNA was isolated using a Power Soil DNA Isolation Kit (MO BIO Laboratories, Carlsbad, CA, USA). Further analysis was performed at ChunLab, Inc. (Seoul, Korea) according to their protocols. Briefly, the concentration of DNA was measured, and the quality of DNA was determined by agarose gel electrophoresis. Bacterial 16S rRNA gene sequences spanning the V3–V4 variable region were amplified using the primer pair 341F_805R. The quality of amplification was confirmed by agarose gel electrophoresis, and the amplicons were subjected to paired-end sequencing using a MiSeq system (Illumina, San Diego, CA, USA). After filtering the low-quality reads and chimeras, an average of approximately 38,017–75,719 reads per group was obtained, which covered ≥99.9% of libraries (Supplementary Table 3). All bioinformatics analyses were performed using software provided in the integrated database EzBioCloud (http://www.ezbiocloud.net/), which is a taxonomically united database of 16S rRNA gene sequences and whole-genome assemblies^38^. The similarity cutoff value for assigning a sequence to the same phylotype was equal to or greater than 97%. When a sequence showed an identity ≥99% with more than one species, it was presented as a taxonomic group represented by the name of species that was identified in the earliest year. However, when all sequence reads in all samples assigned to a specific group showed a top hit with only one species among the species grouped together, the name of the species with top hit was used instead of the group name. The overall phylogenetic distance between communities was estimated using the generalized UniFrac distances and was visualized using PCoA.

### Transcriptome analysis of the salivary glands

RNA purity and integrity were evaluated by an ND-1000 spectrophotometer (NanoDrop, Wilmington, USA) and an Agilent 2100 Bioanalyzer (Agilent Technologies, Palo Alto, USA), respectively. Total RNA samples obtained from 20-week-old noncohoused *Nfkbiz*^+/+^ and *Nfkbiz*^*−*/−^ mice (*n* = 2 per group) were subjected to cDNA synthesis and labeling using a GeneChip™ WT PLUS Reagent Kit (ThermoFisher Scientific) and then to transcriptome analysis using Affymetrix whole-transcript expression arrays (ThermoFisher Scientific) at Macrogen Inc. (Seoul, Korea). The array process was executed according to the manufacturer’s protocol. The data were summarized and normalized with the robust multiaverage method implemented in Affymetrix® Power Tools. After exporting the results with gene-level robust multiaverage analysis, DEG analysis was performed.

### qRT-PCR

Total RNA (1 μg) from the salivary gland was subjected to reverse transcription with oligo dT and reverse transcriptase (Promega, Madison, WI, USA) in a 30-μl reaction mix at 42°C for 1 h. PCR was performed in a 20-μl reaction mix containing 2 μl of template cDNA, SYBR Premix Ex *Taq*, ROX Reference Dye (Takara Bio, Otsu, Japan), and each primer (0.2 μM). Amplification was performed in a fluorescence thermocycler (Applied Biosystems, Foster City, CA, USA) under the following conditions: an initial denaturation at 95 °C for 5 min, followed by 40 cycles of denaturation at 95 °C for 10 s, annealing at 60 °C for 40 s, and elongation at 72 °C for 40 s. The specificity of the PCR product was verified by melting curve analysis and examination on a 3% agarose gel. The housekeeping gene glyceraldehyde-3-phosphate dehydrogenase (*Gapdh*) was amplified in parallel with *Jchain*. The relative copy numbers of *Jchain* compared to those of *Gapdh* were calculated using 2^−∆Ct^. The copy numbers of the 16S rRNA genes of bacteria were estimated using a standard curve that was generated using the genomic DNA of *Porphyromonas gingivalis* ATCC 33277 and expressed as the numbers per 1 μg RNA. The primer sequences used were as follows: 5′- CTTGTAACAGGTGACGACGAAG-3′ and 5′-CCACTTCCACAGGATCGCAT-3′ for *Jchain*, 5′-GTTGTCTCCTGCGACTTCA-3′ and 5′-GGTGGTCCAGGGTTTCTTA-3′ for *Gapdh*, and 5′-AGTCACTGACGAGTTTGATCMTGGCTCAG-3′ and 5′-CAGTGACTACWTTACCGCGGCTGCTGG-3′ for the 16S rRNA gene. PCR was performed in triplicate for each RNA sample.

### ELISA

The concentrations of IgA in the saliva samples were determined in duplicate using a Mouse IgA ELISA Kit (MyBioSource, San Diego, CA, USA) according to the manufacturer’s protocol. Dilutions of 1:10 and 1:20 were used for the samples obtained at week 3 and week 20, respectively. To correct the influence of reduced salivary flow rate on the concentrations of sIgA, data were expressed as total amounts of sIgA calculated by multiplying the concentrations of sIgA (ng/ml) and the weight (g) of saliva collected.

### Statistical analysis

Most data sets obtained from mice were not normally distributed; thus, nonparametric approaches were used. Differences between two groups were determined using the Mann–Whitney *U*-test. Differences between weeks 3 and 20 were determined using the Wilcoxon signed rank test. Differences among four groups were determined using the Kruskal–Wallis test, followed by the Bonferroni post hoc method. Differences between two groups in microarray data were determined using independent *t*-tests. To determine the correlation between two variables, Spearman’s rank correlation test was used. The taxa associated with FLS were determined using LEfSe provided online (https://huttenhower.sph.harvard.edu/galaxy/). The false discovery rate was controlled by adjusting the *P* value using the Benjamini–Hochberg algorithm by using R 3.3.2 (www.r-project.org). PERMANOVA was performed with the Adonis function from the vegan package in R. All other statistical analyses were performed using SPSS software version 23.0 (IBM, Chicago, IL, USA). All tests were two-sided, and statistical significance was set at *P* < 0.05.

### Reporting summary

Further information on experimental design is available in the Nature Research Reporting Summary linked to this paper.

## Supplementary information

Supplementary Information

Reporting Summary

## Data Availability

The sequence data of the oral microbiota can be accessed at the NCBI Sequence Read Archive under the accession number PRJNA560734). The transcriptome data of the salivary glands can be accessed at the NCBI Gene Expression Omnibus under the accession number GSE151163. The processed data of the oral microbiota and transcriptome analyses are available from the corresponding author upon request.

## Code availability

Custom software that has not been published was not used in this study.
