# Supplementary material for: Association of a dysbiotic oral microbiota with the development of focal lymphocytic sialadenitis in IκB-ζ-deficient mice
Source: NPJ Biofilms Microbiomes. 2020 Oct 30;6:49. doi: 10.1038/s41522-020-00158-4 (PMC7599236; doi:10.1038/s41522-020-00158-4)
Supplement: Supplementary file 1 — Supplementary Information [file 41522_2020_158_MOESM1_ESM.pdf]

Supplementary Table 1. Mice and cage ID used in the study

Age	Mouse ID	Genotype	Cage ID[a]	Housing Condition
week 24	M-19	*Nfkbiz*$^{-/-}$	1	non-cohoused
	M-25	*Nfkbiz*$^{-/-}$	2	non-cohoused
	M-0	*Nfkbiz*$^{+/+}$	3	non-cohoused
	M-45			
	M-49			
	M-50			
	M-51			
	M-82	*Nfkbiz*$^{-/-}$	4	non-cohoused
	M-183	*Nfkbiz*$^{-/-}$	5	non-cohoused
	M-188			
week 3 & week 20	M40	*Nfkbiz*$^{-/-}$	6	cohoused
	M28	*Nfkbiz*$^{+/+}$		
	M29			
	M31	*Nfkbiz*$^{+/+}$	7	non-cohoused
	M32			
	M47	*Nfkbiz*$^{-/-}$	8	non-cohoused
	M48			
	M49			
	M53	*Nfkbiz*$^{-/-}$	9	cohoused
	M57	*Nfkbiz*$^{+/+}$		
	M55	*Nfkbiz*$^{+/+}$	10	non-cohoused
	M56			
	M58			
	M59			
	M74	*Nfkbiz*$^{-/-}$	11	cohoused
	M76			
	M79	*Nfkbiz*$^{+/+}$		
	M82	*Nfkbiz*$^{-/-}$	12	cohoused
	M83	*Nfkbiz*$^{+/+}$		
	M88	*Nfkbiz*$^{-/-}$	13	non-cohoused
	M89			
	M207	*Nfkbiz*$^{+/+}$	14	cohoused
	M213	*Nfkbiz*$^{-/-}$		
	M231	*Nfkbiz*$^{-/-}$	15	non-cohoused

[a]The cage ID is in a chronological order based on the date of birth.

Supplementary Table 2. Differentially expressed genes in the salivary glands of *Nfkbiz*$^{-/-}$ vs. *Nfkbiz*$^{+/+}$ mice.

Gene_Symbol	Description	KO/WT fold change	KO/WT volume	*p*	*q*
Jchain	immunoglobulin joining chain	7.36	4.74	0.017	0.957
Oas1a	2-5 oligoadenylate synthetase 1A	3.79	4.12	0.003	0.957
Usp18	ubiquitin specific peptidase 18	3.19	6.02	0.045	0.957
Spi1	spleen focus forming virus (SFFV) proviral integration oncogene	2.82	6.24	0.029	0.957
Znfx1	zinc finger, NFX1-type containing 1	2.75	5.88	0.023	0.957
Lgals9	lectin, galactose binding, soluble 9	2.55	5.52	0.021	0.957
Baiap2	brain-specific angiogenesis inhibitor 1-associated protein 2	2.37	5.60	0.011	0.957
Eif2ak2	eukaryotic translation initiation factor 2-alpha kinase 2	2.34	6.90	0.006	0.957
Xaf1	XIAP associated factor 1	2.25	4.95	0.044	0.957
Insc	Inscuteable homolog	2.23	3.43	0.011	0.957
Sdc3	syndecan 3	2.23	5.46	0.037	0.957
H2-Q7	histocompatibility 2, Q region locus 7	2.20	7.63	0.046	0.957
Fn1	fibronectin 1	2.15	5.69	0.007	0.957
Elk4	ELK4, member of ETS oncogene family	2.15	7.00	0.007	0.957
Apbb2	amyloid beta (A4) precursor protein-binding, family B, member 2	2.13	8.10	0.040	0.957
H2-K1	histocompatibility 2, K1, K region	2.03	10.24	0.039	0.957
Ino80	INO80 homolog	2.01	6.40	0.032	0.957
Chd7	chromodomain helicase DNA binding protein 7	1.98	7.76	0.037	0.957
Ccr2	chemokine (C-C motif) receptor 2	1.98	2.62	0.035	0.957
Slc44a5	solute carrier family 44, member 5	1.94	1.82	0.029	0.957
Fndc3b	fibronectin type III domain	1.92	8.63	0.024	0.957

	containing 3B				
Mast4	microtubule associated serine/threonine kinase family member 4	1.90	8.08	0.043	0.957
Magea6	melanoma antigen, family A, 6	1.89	2.82	0.030	0.957
Mir23b	microRNA 23b	1.89	7.70	0.033	0.957
Zbtb20	zinc finger and BTB domain containing 20	1.89	8.28	0.013	0.957
Tnfrsf1b	tumor necrosis factor receptor superfamily, member 1b	1.88	5.03	0.035	0.957
Iqcf3	IQ motif containing F3	1.87	3.06	0.005	0.957
Prelp	proline arginine-rich end leucine-rich repeat	1.87	5.57	0.010	0.957
Tlr7	toll-like receptor 7	1.86	5.47	0.018	0.957
Ambra1	autophagy/beclin 1 regulator 1	1.86	8.23	0.046	0.957
Rasal1	RAS protein activator like 1	1.83	7.08	0.019	0.957
Sin3a	transcriptional regulator, SIN3A	1.82	8.39	0.026	0.957
Nktr	natural killer tumor recognition sequence	1.82	8.64	0.032	0.957
Fign	fidgetin	1.82	3.60	0.014	0.957
Rptor	regulatory associated protein of MTOR, complex 1	1.82	6.36	0.038	0.957
Lrif1	ligand dependent nuclear receptor interacting factor 1	1.82	5.16	0.011	0.957
Scaf4	SR-related CTD-associated factor 4	1.81	7.95	0.023	0.957
Uvrag	UV radiation resistance associated gene	1.79	6.39	0.005	0.957
Clec4g	C-type lectin domain family 4, member g	1.78	3.94	0.030	0.957
Pld4	phospholipase D family, member 4	1.78	5.85	0.030	0.957
Clip4	CAP-GLY domain containing linker protein family, member 4	1.78	7.84	0.018	0.957
Atxn7	ataxin 7	1.76	6.72	0.020	0.957
Gm11042	predicted gene 11042 [Source:MGI Symbol;Acc:MGI:3779266]	1.76	5.23	0.025	0.957

Gm10217	predicted gene 10217 [Source:MGI Symbol;Acc:MGI:3642099]	1.75	10.50	0.032	0.957
Dennd1a	DENN/MADD domain containing 1A	1.75	6.04	0.042	0.957
Map3k13	mitogen-activated protein kinase kinase kinase 13	1.74	5.56	0.029	0.957
Ghrl	ghrelin	1.74	5.68	0.039	0.957
Grn	granulin	1.73	9.13	0.046	0.957
Arhgap26	Rho GTPase activating protein 26	1.72	7.67	0.021	0.957
Traf3ip3	TRAF3 interacting protein 3	1.70	4.55	0.027	0.957
Exoc6b	exocyst complex component 6B	1.70	6.55	0.005	0.957
Kmt2a	lysine (K)-specific methyltransferase 2A	1.70	7.02	0.040	0.957
Pomt1	protein-O-mannosyltransferase 1	1.69	6.93	0.033	0.957
Pik3ap1	phosphoinositide-3-kinase adaptor protein 1	1.69	7.30	0.005	0.957
Olfr692	olfactory receptor 692	1.69	4.06	0.040	0.957
Atp10a	ATPase, class V, type 10A	1.68	4.58	0.024	0.957
Clec3a	C-type lectin domain family 3, member a	1.68	2.73	0.033	0.957
Gm17571	predicted gene, 17571 [Source:MGI Symbol;Acc:MGI:4937205]	1.67	10.93	0.018	0.957
Gbp9	guanylate-binding protein 9	1.67	3.12	0.048	0.957
Arhgap39	Rho GTPase activating protein 39	1.67	6.40	0.028	0.957
Arhgap15	Rho GTPase activating protein 15	1.66	3.68	0.027	0.957
Gm6455	predicted gene 6455	1.65	2.97	0.028	0.957
Tlr9	toll-like receptor 9	1.65	3.94	0.040	0.957
Casz1	castor zinc finger 1	1.65	6.81	0.005	0.957
Zfp248	zinc finger protein 248	1.65	4.61	0.035	0.957
Galnt9	UDP-N-acetyl-alpha-D-galactosamine:polypeptide N-acetylgalactosaminyltransferase 9	1.64	3.89	0.019	0.957
Strada	STE20-related kinase adaptor alpha	1.64	3.79	0.048	0.957
Zfp280c	zinc finger protein 280C	1.64	6.25	0.042	0.957

Efcab9	EF-hand calcium binding domain 9	1.64	2.93	0.014	0.957
Ggcx	gamma-glutamyl carboxylase	1.63	8.20	0.045	0.957
Thbs3	thrombospondin 3	1.62	3.75	0.035	0.957
Clasp1	CLIP associating protein 1	1.62	6.72	0.025	0.957
Zc3h7a	zinc finger CCCH type containing 7 A	1.61	7.04	0.000	0.957
Arsb	arylsulfatase B	1.61	7.44	0.040	0.957
Hdac8	histone deacetylase 8	1.61	5.96	0.028	0.957
Tonsl	tonsoku-like, DNA repair protein	1.60	3.97	0.049	0.957
Hmgxb3	HMG box domain containing 3	1.60	6.28	0.029	0.957
Olfr102	olfactory receptor 102	1.59	1.90	0.016	0.957
Gm3414	predicted gene 3414	1.59	4.53	0.011	0.957
Olfr786	olfactory receptor 786	1.58	2.44	0.033	0.957
Cyp2s1	cytochrome P450, family 2, subfamily s, polypeptide 1	1.58	8.64	0.045	0.957
Dpp7	dipeptidylpeptidase 7	1.58	6.51	0.043	0.957
Med27	mediator complex subunit 27	1.58	6.26	0.024	0.957
Cabyr	calcium-binding tyrosine-(Y)-phosphorylation regulated (fibrousheathin 2)	1.57	3.11	0.025	0.957
Secisbp2	SECIS binding protein 2	1.57	6.06	0.010	0.957
Cdk17	cyclin-dependent kinase 17	1.57	6.28	0.000	0.957
Wdr7	WD repeat domain 7	1.57	6.78	0.021	0.957
Vwc2	von Willebrand factor C domain containing 2	1.57	3.74	0.027	0.957
Scd1	stearoyl-Coenzyme A desaturase 1	1.57	10.09	0.008	0.957
Gpr153	G protein-coupled receptor 153	1.57	3.76	0.040	0.957
Olfr987	olfactory receptor 987	1.56	2.39	0.030	0.957
Dnajb13	DnaJ (Hsp40) related, subfamily B, member 13	1.56	3.33	0.047	0.957
Nvl	nuclear VCP-like	1.55	6.60	0.044	0.957
Bnc1	basonuclin 1	1.55	4.35	0.047	0.957
Ndst1	N-deacetylase/N-sulfotransferase (heparan glucosaminyl) 1	1.54	8.83	0.003	0.957

G530012D18Rik	RIKEN cDNA G530012D1 gene [Source:MGI Symbol;Acc:MGI:3642025]	1.53	3.90	0.019	0.957
Ces1a	carboxylesterase 1A	1.53	3.13	0.013	0.957
Gm11060	predicted gene 11060 [Source:MGI Symbol;Acc:MGI:3779284]	1.53	8.03	0.014	0.957
Cul7	cullin 7	1.52	5.55	0.040	0.957
F2rl2	coagulation factor II (thrombin) receptor-like 2	1.52	4.81	0.041	0.957
Psg20	pregnancy-specific glycoprotein 20	1.52	4.60	0.030	0.957
Gtpbp8	GTP-binding protein 8 (putative)	1.52	4.83	0.041	0.957
Tcf4	transcription factor 4	1.52	8.43	0.014	0.957
Znrf3	zinc and ring finger 3	1.51	7.01	0.036	0.957
Gm694	predicted gene 694	1.51	3.52	0.015	0.957
Shisa5	shisa family member 5	1.50	7.09	0.049	0.957
Gbf1	golgi-specific brefeldin A-resistance factor 1	1.50	9.12	0.046	0.957
Sohlh2	spermatogenesis and oogenesis specific basic helix-loop-helix 2	-1.50	2.36	0.009	0.957
Stub1	STIP1 homology and U-Box containing protein 1	-1.50	6.62	0.042	0.957
Snrpf	small nuclear ribonucleoprotein polypeptide F	-1.51	5.37	0.006	0.957
Mapk1	mitogen-activated protein kinase 1	-1.52	6.51	0.014	0.957
1700029H14Rik	RIKEN cDNA 1700029H14 gene	-1.52	6.04	0.043	0.957
Hint1	histidine triad nucleotide binding protein 1	-1.52	7.33	0.015	0.957
Nme2	NME/NM23 nucleoside diphosphate kinase 2	-1.53	8.13	0.036	0.957
Ppp1r11	protein phosphatase 1, regulatory (inhibitor) subunit 11	-1.53	5.86	0.007	0.957
Ipp	IAP promoted placental gene	-1.53	3.60	0.025	0.957
Dcpp3	demilune cell and parotid protein 3	-1.53	12.55	0.038	0.957
Ptov1	prostate tumor over expressed gene 1	-1.53	7.23	0.016	0.957

Yif1a	Yip1 interacting factor homolog A (S. cerevisiae)	-1.53	5.64	0.046	0.957
2410015M20Rik	RIKEN cDNA 2410015M20 gene	-1.53	6.39	0.046	0.957
Rps5	ribosomal protein S5	-1.54	7.61	0.039	0.957
Mpi	mannose phosphate isomerase	-1.54	7.17	0.024	0.957
Nap1l1	nucleosome assembly protein 1-like 1	-1.54	7.39	0.005	0.957
Dynlt1b	dynein light chain Tctex-type 1B	-1.55	6.48	0.048	0.957
Crygn	crystallin, gamma N	-1.55	5.18	0.015	0.957
Tbx5	T-box 5	-1.55	3.85	0.034	0.957
Cmc2	COX assembly mitochondrial protein 2	-1.55	5.19	0.040	0.957
Angptl4	angiopoietin-like 4	-1.56	4.94	0.041	0.957
Olfr1377	olfactory receptor 1377	-1.56	3.29	0.020	0.957
Nt5c	5,3-nucleotidase, cytosolic	-1.56	5.44	0.028	0.957
Nsmce4a	non-SMC element 4 homolog A (S. cerevisiae)	-1.56	5.54	0.045	0.957
Znhit6	zinc finger, HIT type 6	-1.56	5.26	0.024	0.957
Dhrs4	dehydrogenase/reductase (SDR family) member 4	-1.57	7.33	0.038	0.957
Rfx4	regulatory factor X, 4	-1.57	3.96	0.006	0.957
B230217C12Rik	RIKEN cDNA B230217C12 gene	-1.57	3.94	0.002	0.957
Prorsd1	prolyl-tRNA synthetase domain containing 1	-1.58	5.22	0.004	0.957
Dynll2	dynein light chain LC8-type 2	-1.58	4.82	0.026	0.957
Tbx2	T-box 2	-1.58	6.06	0.037	0.957
Cbx3	chromobox 3	-1.58	8.12	0.026	0.957
Sat2	spermidine/spermine N1-acetyl transferase 2	-1.59	4.18	0.009	0.957
Rps20	ribosomal protein S20	-1.59	7.11	0.042	0.957
Snx1	sorting nexin 1	-1.59	4.98	0.019	0.957
Eif3i	eukaryotic translation initiation factor 3, subunit I	-1.59	6.22	0.036	0.957
Dcpp2	demilune cell and parotid protein 2	-1.59	12.10	0.043	0.957
Rpl19	ribosomal protein L19	-1.59	8.65	0.033	0.957

Tlx1	T cell leukemia, homeobox 1	-1.59	6.56	0.012	0.957
Atp5f1	ATP synthase, H+ transporting, mitochondrial F0 complex, subunit B1	-1.60	7.58	0.008	0.957
St3gal5	ST3 beta-galactoside alpha-2,3-sialyltransferase 5	-1.60	5.64	0.033	0.957
Adrb3	adrenergic receptor, beta 3	-1.60	4.03	0.019	0.957
Igbp1	immunoglobulin (CD79A) binding protein 1	-1.60	6.85	0.046	0.957
Hsdl2	hydroxysteroid dehydrogenase like 2	-1.61	5.01	0.006	0.957
Gm19659	PREDICTED: predicted gene, 19659 (Gm19659), mRNA.	-1.61	1.94	0.038	0.957
Cklf	chemokine-like factor	-1.61	4.35	0.033	0.957
Ppip5k2	diphosphoinositol pentakisphosphate kinase 2	-1.61	5.64	0.029	0.957
Lipm	lipase, family member M	-1.62	2.31	0.004	0.957
Gnb2l1	guanine nucleotide binding protein (G protein), beta polypeptide 2 like 1	-1.63	8.08	0.033	0.957
Mrpl22	mitochondrial ribosomal protein L22	-1.64	3.66	0.016	0.957
Il1r2	interleukin 1 receptor, type II	-1.64	4.28	0.030	0.957
Adck3	aarF domain containing kinase 3	-1.64	5.95	0.041	0.957
Bcl2a1d	B cell leukemia/lymphoma 2 related protein A1d	-1.64	3.55	0.049	0.957
Fgf13	fibroblast growth factor 13	-1.65	2.66	0.040	0.957
Rps26	ribosomal protein S26	-1.65	7.57	0.005	0.957
Aldh7a1	aldehyde dehydrogenase family 7, member A1	-1.65	8.15	0.041	0.957
Gm20736	predicted gene, 20736	-1.65	1.85	0.031	0.957
Gucy1a3	guanylate cyclase 1, soluble, alpha 3	-1.65	3.07	0.028	0.957
Hspa2	heat shock protein 2	-1.65	3.76	0.039	0.957
Serp1	stress-associated endoplasmic reticulum protein 1	-1.66	9.53	0.039	0.957
Glrx3	glutaredoxin 3	-1.66	6.66	0.047	0.957
Suclg1	succinate-CoA ligase, GDP-	-1.66	7.37	0.036	0.957

	forming, alpha subunit				
Veph1	ventricular zone expressed PH domain-containing 1	-1.66	3.21	0.028	0.957
Arl1	ADP-ribosylation factor-like 1	-1.66	8.99	0.017	0.957
Neu2	neuraminidase 2	-1.67	5.79	0.032	0.957
Aven	apoptosis, caspase activation inhibitor	-1.67	5.80	0.015	0.957
Tcf19	transcription factor 19	-1.67	4.16	0.002	0.957
Rpsa	ribosomal protein SA	-1.67	8.56	0.014	0.957
Mtif3	mitochondrial translational initiation factor 3	-1.67	4.60	0.032	0.957
Dynlt1c	dynein light chain Tctex-type 1C	-1.68	5.79	0.023	0.957
Fam174a	family with sequence similarity 174, member A	-1.68	5.97	0.005	0.957
BC031181	cDNA sequence BC031181	-1.69	6.58	0.021	0.957
Tmem42	transmembrane protein 42	-1.70	4.98	0.012	0.957
Hist1h2ac	histone cluster 1, H2ac	-1.70	5.74	0.012	0.957
Pfdn1	prefoldin 1	-1.72	5.68	0.009	0.957
Rps9	ribosomal protein S9	-1.73	7.48	0.015	0.957
Ywhaq	tyrosine 3-monooxygenase/tryptophan 5-monooxygenase activation protein, theta polypeptide	-1.73	7.40	0.037	0.957
Vdac1	voltage-dependent anion channel 1	-1.73	7.44	0.042	0.957
Adprh	ADP-ribosylarginine hydrolase	-1.75	4.68	0.033	0.957
C1ql4	complement component 1, q subcomponent-like 4	-1.76	2.69	0.040	0.957
Hibadh	3-hydroxyisobutyrate dehydrogenase	-1.77	7.53	0.041	0.957
Hsbp1l1	heat shock factor binding protein 1-like 1	-1.77	4.95	0.046	0.957
Dpm3	dolichyl-phosphate mannosyltransferase polypeptide 3	-1.78	6.83	0.032	0.957
Ndufa9	NADH dehydrogenase (ubiquinone) 1 alpha subcomplex, 9	-1.78	6.95	0.009	0.957

Fbxw9	F-box and WD-40 domain protein 9	-1.78	4.85	0.016	0.957
Ntan1	N-terminal Asn amidase	-1.80	5.51	0.004	0.957
Rps4x	ribosomal protein S4, X-linked	-1.80	7.37	0.038	0.957
Abhd3	abhydrolase domain containing 3	-1.81	4.61	0.011	0.957
Tnnc2	troponin C2, fast	-1.83	4.59	0.036	0.957
Blvra	biliverdin reductase A	-1.83	5.22	0.015	0.957
Ccl28	chemokine (C-C motif) ligand 28	-1.83	9.24	0.039	0.957
Hint2	histidine triad nucleotide binding protein 2	-1.85	4.40	0.040	0.957
Ube2a	ubiquitin-conjugating enzyme E2A	-1.85	4.68	0.014	0.957
Cnpy4	canopy 4 homolog (zebrafish)	-1.87	4.01	0.020	0.957
Anapc4	anaphase promoting complex subunit 4	-1.87	4.56	0.045	0.957
Adgrg3	adhesion G protein-coupled receptor G3	-1.87	3.62	0.044	0.957
Moap1	modulator of apoptosis 1)	-1.88	3.58	0.001	0.957
Ndufa6	NADH dehydrogenase (ubiquinone) 1 alpha subcomplex, 6 (B14)	-1.96	6.20	0.023	0.957
Tpt1	tumor protein, translationally-controlled 1	-1.98	9.19	0.014	0.957
Acp1	acid phosphatase 1, soluble	-1.98	5.49	0.030	0.957
Anxa2	annexin A2	-1.99	5.43	0.038	0.957
Etfa	electron transferring flavoprotein, alpha polypeptide	-1.99	7.52	0.045	0.957
Gnmt	glycine N-methyltransferase	-2.00	6.57	0.019	0.957
Insig1	insulin induced gene 1	-2.21	8.25	0.028	0.957
Rpl36al	ribosomal protein L36A-like	-2.63	6.50	0.044	0.957

Supplementary Table 3. The summary of sequencing characteristics

	Number of raw reads	Number of valid reads	Average read length	Coverage %
24 week old *Nfkbiz*$^{+/+}$ (n=5)	76,586 ± 10,119	63,527 ± 8,614	426 ± 1.5	99.97 ± 0.01
24 week old *Nfkbiz*$^{-/-}$ (n=5)	88,757 ± 14,443	72,844 ± 12,437	425 ± 1.1	99.97 ± 0.01
3 week old non-cohoused *Nfkbiz*$^{+/+}$ (n=6)	40,673 ± 15,750	37,962 ± 15,032	425 ± 2.2	99.94 ± 0.05
3 week old cohoused *Nfkbiz*$^{+/+}$ (n=6)	67,115 ± 18,277	52,360 ± 18,837	425 ± 1.5	99.91 ± 0.09
3 week old cohoused *Nfkbiz*$^{-/-}$ (n=6)	75,172 ± 25,495	65,805 ± 21,319	425 ± 0.8	99.93 ± 0.07
3 week old non-cohoused *Nfkbiz*$^{-/-}$ (n=6)	77,651 ± 31,487	67,109 ± 22,892	426 ± 0.4	99.96 ± 0.01
20 week old non-cohoused *Nfkbiz*$^{+/+}$ (n=6)	47,015 ± 13,615	43,618 ± 12,109	426 ± 0.5	99.93 ± 0.01
20 week old cohoused *Nfkbiz*$^{+/+}$ (n=6)	54,692 ± 43,660	43,526 ± 25,561	426 ± 1.0	99.89 ± 0.08
20 week old cohoused *Nfkbiz*$^{-/-}$ (n=6)	48,650 ± 21,020	44,026 ± 19,188	426 ± 0.6	99.93 ± 0.05
20 week old non-cohoused *Nfkbiz*$^{-/-}$ (n=6)	51,059 ± 30,985	44,182 ± 26,252	426 ± 0.9	99.93 ± 0.05

```
NR_117375.1   315 AGGGAATCTTCGGCAATGGGGGCAACCCTGACCGAGCAACGCCGCGTGAGTGAAGAAGGT  374
Current study   1 ............................................................  60

NR_117375.1   375 TTTCGGATCGTAAAGCTCTGTTGTAAGAGAAGAACGTGTGTGAGAGTGGAAAGTTCACAC  434
Current study  61 ............................................................  120

NR_117375.1   435 AGTGACGGTAACTTACCAGAAAGGGACGGCTAACTACGTGCCAGCAGCCGCGGTAATACG  494
Current study 121 ............................................................  180

NR_117375.1   495 TAGGTCCCGAGCGTTATCCGGATTTATTGGGCGTAAAGCGAGCGCAGGCGGTTGGATAAG  554
Current study 181 ............................................................  240

NR_117375.1   555 TCTGAAGTTAAAGGCTGTGGCTCAACCATAGTATGCTTTGGAAACTGTTCAACTTGAGTG  614
Current study 241 ......................T.....................................  300

NR_117375.1   615 CAGAAGGGGAGAGTGGAATTCCATGTGTAGCGGTGGAATGCGTAGATATATGGAGGAACA  674
Current study 301 ............................................................  360

NR_117375.1   675 CCGGTGGCGAAAGCGGCTCTCTGGTCTGTAACTGACGCTGAGGCTCGAAAGCGTGGGTAG  734
Current study 361 ............................................................  420

NR_117375.1   735 CGAAC  739
Current study 421 .....  425
```

Supplementary Figure 1. The sequence of 16S rRNA gene of *Streptococcus danieliae* The sequence of a dominant amplified sequence variant assigned to *S. danieliae* in the current study was compared to the sequence of 16S rRNA gene of *S. danieliae* strain ERD01G reported by Clavel et al.[21]
