# Supplementary material for: Association of a dysbiotic oral microbiota with the development of focal lymphocytic sialadenitis in IκB-ζ-deficient mice
Source: NPJ Biofilms Microbiomes. 2020 Oct 30;6:49. doi: 10.1038/s41522-020-00158-4 (PMC7599236; doi:10.1038/s41522-020-00158-4)
Supplement: Supplementary file 2 — Reporting Summary [file 41522_2020_158_MOESM2_ESM.pdf]

nature research

Corresponding author(s): Youngnim Choi

Last updated by author(s): Sep 18, 2020

Reporting Summary

Nature Research wishes to improve the reproducibility of the work that we publish. This form provides structure for consistency and transparency in reporting. For further information on Nature Research policies, see our Editorial Policies and the Editorial Policy Checklist.

Statistics

For all statistical analyses, confirm that the following items are present in the figure legend, table legend, main text, or Methods section.

n/a	Confirmed	
☐	☒	The exact sample size (*n*) for each experimental group/condition, given as a discrete number and unit of measurement
☐	☒	A statement on whether measurements were taken from distinct samples or whether the same sample was measured repeatedly
☐	☒	The statistical test(s) used AND whether they are one- or two-sided<br>*Only common tests should be described solely by name; describe more complex techniques in the Methods section.*
☒	☐	A description of all covariates tested
☐	☒	A description of any assumptions or corrections, such as tests of normality and adjustment for multiple comparisons
☐	☒	A full description of the statistical parameters including central tendency (e.g. means) or other basic estimates (e.g. regression coefficient) AND variation (e.g. standard deviation) or associated estimates of uncertainty (e.g. confidence intervals)
☐	☒	For null hypothesis testing, the test statistic (e.g. *F*, *t*, *r*) with confidence intervals, effect sizes, degrees of freedom and *P* value noted<br>*Give P values as exact values whenever suitable.*
☒	☐	For Bayesian analysis, information on the choice of priors and Markov chain Monte Carlo settings
☒	☐	For hierarchical and complex designs, identification of the appropriate level for tests and full reporting of outcomes
☐	☒	Estimates of effect sizes (e.g. Cohen's *d*, Pearson's *r*), indicating how they were calculated

Our web collection on statistics for biologists contains articles on many of the points above.

Software and code

Policy information about availability of computer code

Data collection	All bioinformatics analyses were performed using software provided in the integrated database EzBioCloud (http://www.ezbiocloud.net/), which is a taxonomically united database of 16S rRNA gene sequences and whole-genome assemblies.
Data analysis	All bioinformatics analyses were performed using software provided in the integrated database EzBioCloud (http://www.ezbiocloud.net/), which is a taxonomically united database of 16S rRNA gene sequences and whole-genome assemblies.

For manuscripts utilizing custom algorithms or software that are central to the research but not yet described in published literature, software must be made available to editors and reviewers. We strongly encourage code deposition in a community repository (e.g. GitHub). See the Nature Research guidelines for submitting code & software for further information.

Data

Policy information about availability of data

All manuscripts must include a data availability statement. This statement should provide the following information, where applicable:

- Accession codes, unique identifiers, or web links for publicly available datasets
- A list of figures that have associated raw data
- A description of any restrictions on data availability

The sequence data of the oral microbiota can be accessed at the NCBI Sequence Read Archive under the accession number PRJNA560734 (https://www.ncbi.nlm.nih.gov/sra/PRJNA560734). The transcriptome data of the salivary glands can be accessed at the NCBI Gene Expression Omnibus under the accession number GSE151163.

Field-specific reporting

Please select the one below that is the best fit for your research. If you are not sure, read the appropriate sections before making your selection.

☒ Life sciences ☐ Behavioural & social sciences ☐ Ecological, evolutionary & environmental sciences

For a reference copy of the document with all sections, see nature.com/documents/nr-reporting-summary-flat.pdf

Life sciences study design

All studies must disclose on these points even when the disclosure is negative.

Sample size

The sample size (n = 5 per group) for the initial study using 24-week-old mice was chosen based on a previous study that reported the lachrymal gland phenotype4. Using the intergroup distance of the oral microbiota and the incidence of FLS score ≥ 1 obtained in an initial study, the sample sizes for the cohousing study were determined to be n = 4 per group to achieve a significant intergroup difference in the oral microbiota based on the simulated PERMANOVA power estimation37 and n = 8 per group to achieve a significant difference in the incidence of FLS score ≥ 1 at ? = 0.05 and 80% power.

Data exclusions

The initial sample size for the cohousing study was set as n = 8 per group. However, five mice were lost during saliva collection or housing due to mishandling. Two mice were not born within the study period. Finally, all data sets were available only for n = 6 per group, and one non-cohoused Nfkbiz[illegible]mouse that was born last was excluded from the analysis.

Replication

Reproducibility of the salivary gland phenotype and oral dysbiosis of Nfkbiz-/- mice was confirmed in two data sets obtained from 24-week-old and 20-week-old mice. However, reproducibility of the cohousing effect on the salivary gland phenotype and oral dysbiosis of Nfkbiz-/- mice was not confirmed, because the cohousing experiment was performed once.

Randomization

Six breeding sets using mice from the same litter were prepared, and Nfkbiz+/+ and Nfkbiz-/- mice born within a 4-day interval were cohoused after weaning. The mice born within an interval of more than 4 days were housed separately by genotype.

Blinding

The Nfkbiz-/- mice have a phenotype on their face. Therefore, it is impossible not to know their genotype upon sampling. Salivary flow rate was determined with the aid of a student who did not know the experimental design. The sections of the salivary glands for the evaluation of focal lymphocytic sialadenitis were named by a mouse number. It is difficult to remember which group each mouse belongs to. The analysis of oral microbiota was done in a commercial company who is blind to the experimental design.

Reporting for specific materials, systems and methods

We require information from authors about some types of materials, experimental systems and methods used in many studies. Here, indicate whether each material, system or method listed is relevant to your study. If you are not sure if a list item applies to your research, read the appropriate section before selecting a response.

Materials & experimental systems

n/a	Involved in the study	
☐	☒	Antibodies
☒	☐	Eukaryotic cell lines
☒	☐	Palaeontology and archaeology
☐	☒	Animals and other organisms
☒	☐	Human research participants
☒	☐	Clinical data
☒	☐	Dual use research of concern

Methods

n/a	Involved in the study	
☒	☐	ChIP-seq
☒	☐	Flow cytometry
☒	☐	MRI-based neuroimaging

Antibodies

Antibodies used

CD3-ζ Antibody (6B10.2), rat anti-mouse B220 clone 553090, goat anti-mouse IgG (H+L) Alexa 488 clone A28175, alkaline phosphatase-conjugated anti-digoxigenin antibody

Validation

CD3-ζ Antibody (6B10.2): https://www.citeab.com/antibodies/809486-sc-1239-cd3-antibody-6b10-2
rat anti-mouse B220 clone 553090: https://www.bdbiosciences.com/us/applications/research/stem-cell-research/hematopoietic-stem-cell-markers/mouse/negative-markers/pe-rat-anti-mouse-cd45rb220-ra3-6b2/p/553090
goat anti-mouse IgG (H+L) Alexa 488 clone A28175: https://www.thermofisher.com/antibody/product/Goat-anti-Mouse-IgG-H-L-Secondary-Antibody-Recombinant-Polyclonal/A28175
alkaline phosphatase-conjugated anti-digoxigenin antibody: https://www.sigmaaldrich.com/content/dam/sigma-aldrich/docs/Roche/Bulletin/1/11093274910bul.pdf

Animals and other organisms

Policy information about studies involving animals; ARRIVE guidelines recommended for reporting animal research

Laboratory animals	Nfkbiz+/+ and Nfkbiz-/- female mice in the 129/Ola x C57BL/6 background, 3 week, 20 week, and 24 week old.
Wild animals	N/A
Field-collected samples	All mice were maintained under specific pathogen-free conditions at 18-23°C with 40-60% humidity and 12 light/12 dark cycle in the Laboratory Animal Facility at the School of Dentistry, Seoul National University. Housing strategy is provided in Supplementary Table 1.
Ethics oversight	The experimental protocols and animal handling procedures were approved by the Seoul National University Animal Care and Use Committee (#SNU-180914-8).

Note that full information on the approval of the study protocol must also be provided in the manuscript.
